# Decarboxylative 1,3-dipolar cycloaddition of amino acids for the synthesis of heterocyclic compounds

**DOI:** 10.3762/bjoc.19.123

**Published:** 2023-11-06

**Authors:** Xiaofeng Zhang, Xiaoming Ma, Wei Zhang

**Affiliations:** 1 Center for Green Chemistry and Department of Chemistry, University of Massachusetts Boston, 100 Morrissey Boulevard, Boston, MA 02125, USA; 2 Department of Medicinal Chemistry, Cerevel Therapeutics, 222 Jacobs St Suite 200, Cambridge, MA 02141, USA; 3 School of Pharmacy, Changzhou University, Changzhou 213164, China

**Keywords:** [3 + 2] cycloaddition, decarboxylation, 1,3-dipolar, double cycloaddition, one-pot synthesis, multicomponent reaction, semi-stabilized azomethine ylide

## Abstract

The [3 + 2] cycloadditions of stabilized azomethine ylides (AMYs) derived from amino esters are well-established. However, the reactions of semi-stabilized AMYs generated from decarboxylative condensation of α-amino acids with arylaldehydes are much less explored. The [3 + 2] adducts of α-amino acids could be used for a second [3 + 2] cycloaddition as well as for other post-condensation modifications. This article highlights our recent work on the development of α-amino acid-based [3 + 2] cycloaddition reactions of *N*–H-type AMYs in multicomponent, one-pot, and stepwise reactions for the synthesis of diverse heterocycles related to some bioactive compounds and natural products.

## Introduction

**Figure 1:**
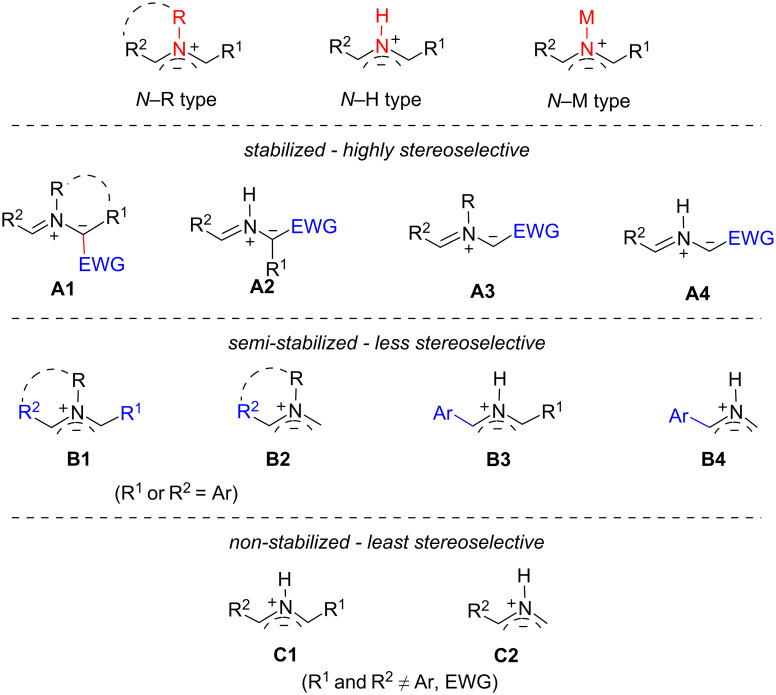
Classification of AMYs.

The 1,3-dipolar cycloaddition of azomethine ylides (AMYs) [1–6] is a powerful method for the synthesis of bioactive pyrrolidine-containing compounds and natural product analogs [7–15]. AMYs generated from the reaction of aldehydes and α-amino esters (via dehydration) or α-amino acids (via decarboxylation) could be classified based on the substitution groups on the N atom to: 1) N-substituted (*N*–R type), 2) hydrogen containing (*N*–H type), and 3) metal complexes (*N*–M type) (Figure 1) [16–17]. These AMYs could also be classified as stabilized (**A1**–**A4**) which contain an electron-withdrawing group (EWG), semi-stabilized (**B1**–**B4**) which have an aryl (Ar) substituent, and non-stabilized (**C1** and **C2**) which have neither an Ar group nor an EWG on the α-carbon atoms.

**Scheme 1:**
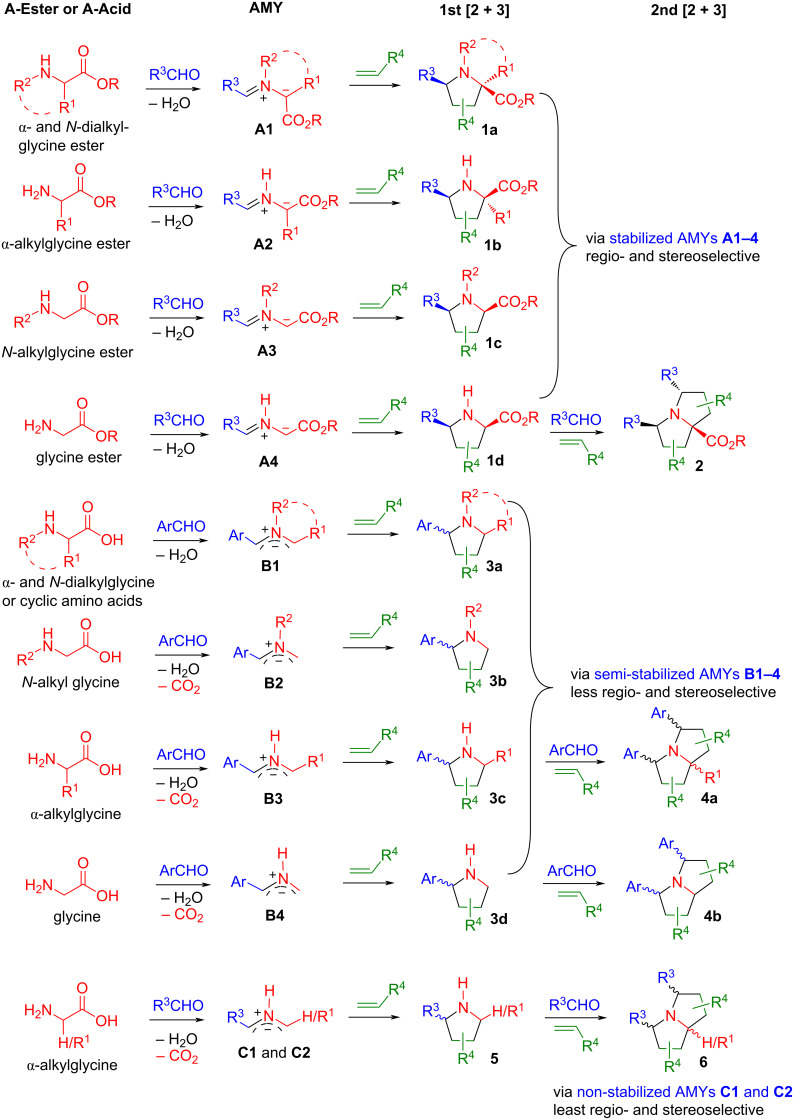
Aminoester- and amino acid-based AMYs for single and double [3+2] cycloadditions.

The routes to access AMYs of different classes are shown in Scheme 1: **A1**-type AMYs can be generated from the condensation of aldehydes with α- and *N*-dialkylglycine esters, **A2**-type AMYs are derived from α-alkylglycine esters, **A3**-type AMYs are derived from *N*-alkylglycine esters, and **A4**-type AMYs are derived from glycine esters. Stabilized zwitterions **A1**–**A4** have the anionic charge on the α-carbon connecting to the EWG. They are popular AMYs for 1,3-diploar [3 + 2] cycloaddition reactions with alkenes to generate pyrrolidines **1a**–**d** with high regio- and stereoselectivities. They have been reported in a huge number (1,000+) of publications [18–28]. It is worth noting that among the products **1a**–**d**, only compound **1d** has hydrogen atoms on both the nitrogen and α-carbon atoms, which makes it suitable to be used for a second cycloaddition to form double [3 + 2] cycloaddition products **2** [29–30].

The *N*–R-type AMYs **B1** and **B2** bearing an Ar group on the α-carbon atom are semi-stabilized (Scheme 1) [16]. The **B1**-type AMYs can be generated from the decarboxylative condensation of aldehydes with α- and *N*-dialkylglycines or from cyclic amino acids (such as proline) [31–33], while AMYs of type **B2** are accessible through the decarboxylative condensation of *N*-dialkylglycines [34–51]. The *N*–H-type semi-stabilized AMYs **B3** are generated through decarboxylative condensation of arylaldehydes with α-alkylglycines, while **B4**-type AMYs are derived from the reaction of glycine [52–58]. The [3 + 2] cycloadditions of AMYs **B1**–**B4** with alkenes lead to the formation of cycloaddition products **3a**–**d** with attenuated regio- and stereoselectivity, since the Ar group is not strong enough to fully localize the negative charge on the carbon connecting to Ar in the 1,3-dipoles. Both products **3c** and **3d** can be used for a second cycloaddition to form products **4a** and **4b**. The non-stabilized AMYs **C1** and **C2** have neither an EWG nor an Ar group to localize the negative charge. The 1,3-dipolar cycloadditions of **C**-type AMYs lead to the formation of [3 + 2] adducts **5** or **6** with low regio- and stereoselectivity which limits the synthetic utility of non-stabilized AMYs of type **C**.

There are over 300 papers on the amino acid-based decarboxylative [3 + 2] cycloadditions of *N*–R-type AMYs **B1** (such as that derived from proline) and **B2** [31–51]. However, to the best of our knowledge, there are only few examples on the reactions of *N*–H-type semi-stabilized AMYs **B3** or **B4** which were either derived from special carbonyl compounds (such as isatin) [52–55] or the AMYs were reacted with uncommon alkenes as the 1,3-dipolarophiles (such as C_60_/C_70_ fullerenes) [56–58].

**Scheme 2:**
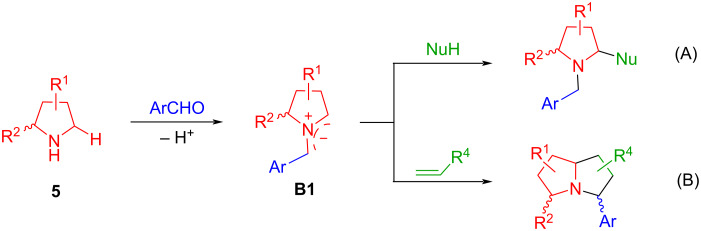
Formation of semi-stabilized AMYs **B1** from pyrrolidines.

**Scheme 3:**
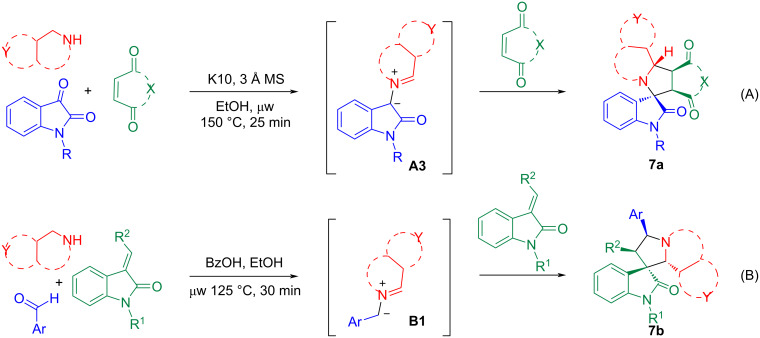
Cyclic amine-based AMYs **A3** and **B1** for [3 + 2] cycloadditions.

Other than amino esters and amino acids shown in Scheme 1, cyclic amines can also react with arylaldehydes to form **B1**-type semi-stabilized AMYs. In this context, the Seidel group reported the reactions of pyrrolidines **5** with arylaldehydes for the formation of AMYs **B1** which then were reacted with nucleophiles to form C–H-functionalized pyrrolidines or subjected to the 1,3-dipolar cycloaddition with olefins to afford bicyclic compounds (Scheme 2A and B) [59–60]. We employed cyclic amines for the synthesis of spirooxindole-pyrrolidines **7a** or **7b** in good stereoselectivity (Scheme 3) [61–62].

**Scheme 4:**
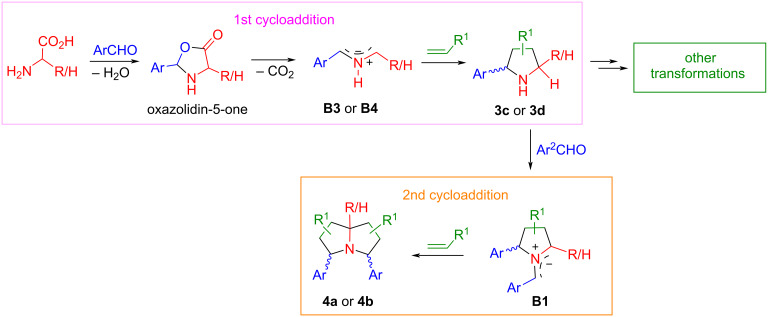
Proposed double cycloaddition reactions involving semi-stabilized AMYs.

From the results shown in Scheme 2 and Scheme 3, we envisioned that pyrrolidines **3c** or **3d** generated from the cycloaddition of AMYs **B3** or **B4** could undergo a second cycloaddition to form double cycloaddition products **4a** or **4b** (Scheme 4). The double cycloaddition process involves two kinds of AMYs, with the first ones (*N*-H-type **B3** or **B4**) derived from amino acids, while the second ones (*N*-R-type **B1**) derived from pyrrolidines **3c** or **3d**. It is worth noting that the double cycloaddition reaction is a pseudo-five-component reaction of amino acids with two equivalents each of aldehydes and alkenes. The first cycloaddition products **3c** or **3d** can also be used as intermediates for other transformations to synthesize novel heterocyclic rings via multicomponent, one-pot, and stepwise synthesis [63–64].

Presented in the following sections is our work on the development of amino acid-based decarboxylative [3 + 2] cycloadditions of *N*–H-type AMYs **B3** and **B4** for double cycloadditions. The stereochemistry of the cycloadditions and the combination of the cycloaddition with other transformations to be one-pot or stepwise reactions are also presented.

## Perspective

### One-step synthesis of trifluoromethylated pyrrolidines

**Scheme 5:**
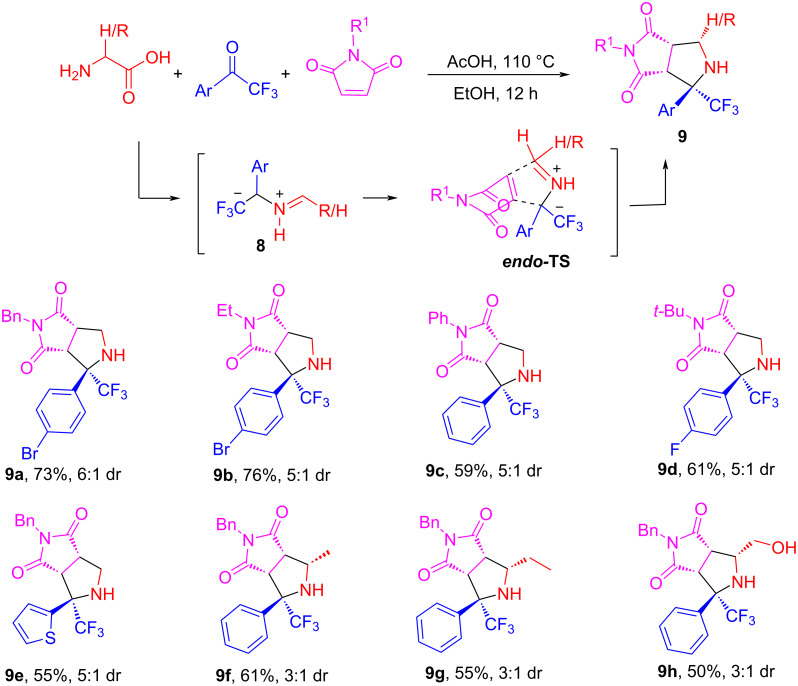
[3 + 2] Cycloaddition for the synthesis of trifluoromethylated pyrrolidines **9**.

As mentioned above, the unexpected double cycloaddition and low stereoselectivity are the major challenges for [3 + 2] cycloaddition reactions of semi- and non-stabilized AMYs derived from the condensation of amino acids with aldehydes. However, the reactions of amino acids with ketones can result in a different kind of AMYs to address the issue. The reaction of trifluoromethyl ketones with glycine or α-substituted amino acids generated stabilized AMY **8** which underwent cycloaddition with maleimides to give 2-CF_3_-substituted pyrrolidines **9** in 50–76% yield (Scheme 5) [65]. Both the Ar and CF_3_ groups can localize the negative charge and also provide steric effects to afford stereoselective cycloaddition products with 3:1 to 6:1 dr. The steric hindrance also prevents products **9** from undergoing a second cycloaddition. The control reactions of methyl ketone or benzaldehydes gave much lower yields and stereoselectivity because of the lacking CF_3_ group. This was the first example of synthesizing 2-CF_3_-substituted pyrrolidines via decarboxylative [3 + 2] cycloaddition which is more efficient than multi-step and metal-assisted syntheses reported in the literature [66–67].

### Pseudo-five-component double cycloadditions for polycyclic pyrrolizidines

**Figure 2:**
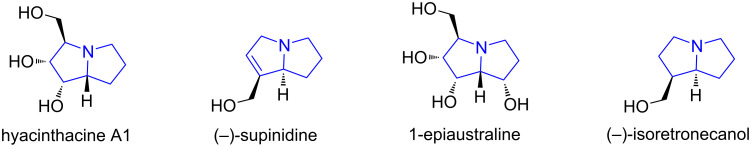
Biologically interesting pyrrolizidines.

With the success of the three-component [3 + 2] cycloadditions shown in Scheme 5, we then explored the double cycloaddition reactions proposed in Scheme 4. The reaction has synthetic significance since the resulting pyrrolizidine scaffold can be found in many biologically active compounds and natural products such as 1-epiaustraline, hyacinthacine A1, (−)-isoretronecanol, and (−)-supinidine (Figure 2) [68–69].

**Scheme 6:**
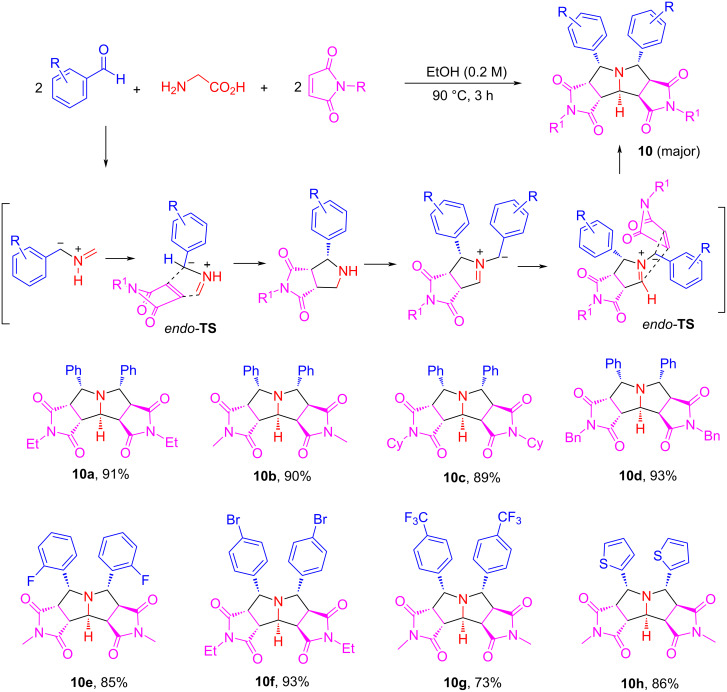
Double cycloadditions with glycine for the synthesis of products **10** (dr > 9:1).

**Scheme 7:**
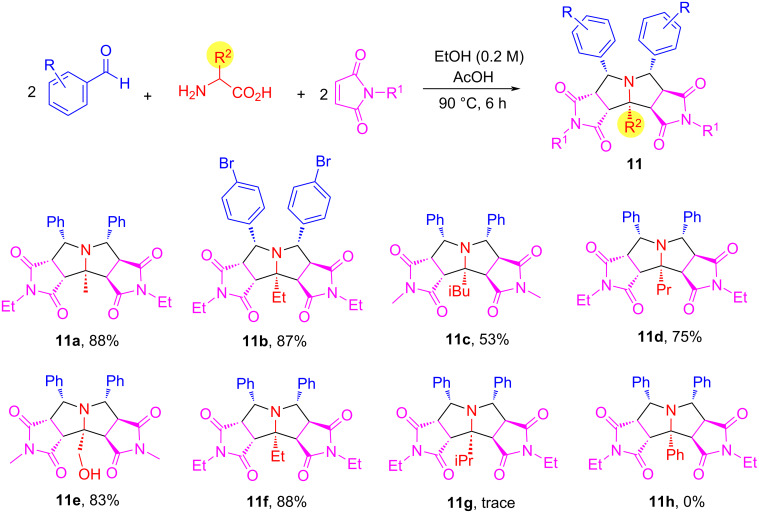
Double cycloadditions with α-substituted amino acids leading to products **11** (≈8.5:1 dr).

After the method development work, a pseudo-five-component double cycloaddition reaction of glycine with two equivalents each of arylaldehydes and *N*-substituted maleimides was carried out in EtOH as a protic solvent at 90 °C for 3 h to afford pyrrolizidines **10** in 73–93% yield with greater than 9:1 dr (Scheme 6). The scope of the reaction could be readily extended for α-substituted amino acids, such as alanine, leucine, serine, and norvaline to give products **11a**–**f** in 53–88% yields with greater than 8.5 dr (Scheme 7). The reactions with leucine and phenylglycine (R^2^ = iPr and Ph) as amino acids gave mainly mono-cycloaddition products and very little double cycloaddition products **11g** and **11h** due to the steric hindrance of the R^2^ group.

**Scheme 8:**
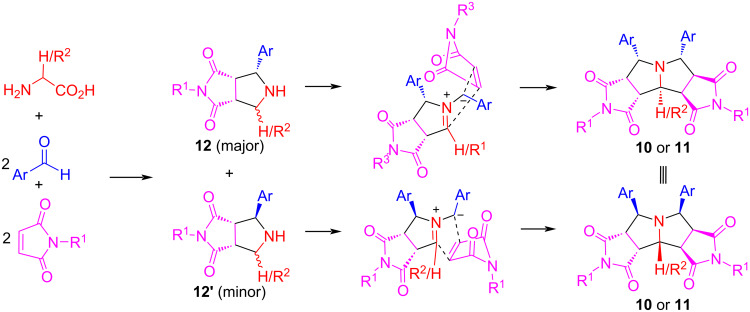
Stereochemistry for the formation of products **10** or **11**.

The stereochemistry of products **10** and **11** was confirmed by X-ray crystal structure and the ^1^H NMR analysis of both the major and minor diastereomers [69]. The first cycloaddition gives adducts **12** and **12’** as a diastereomeric mixture. At the second cycloaddition, both major and minor adducts from the first cycloaddition generate the same products **10** or **11** (Scheme 8).

**Scheme 9:**
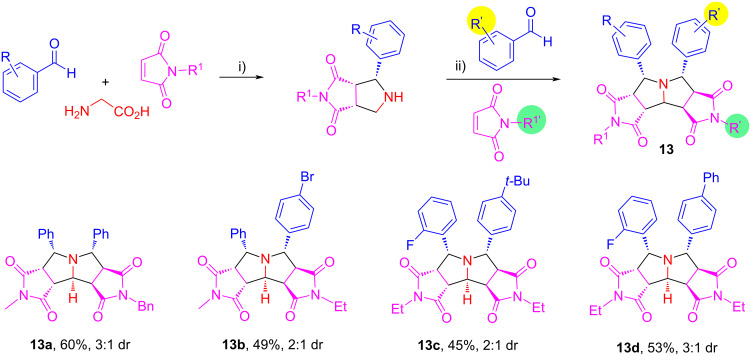
One-pot and stepwise double cycloadditions. Conditions: i) MeCN (0.02 M), 90 °C, 6 h; ii) then AcOH (0.5 equiv), 125 °C, 12 h.

We also evaluated the double cycloadditions in two operational steps by using two different sets of aldehydes and maleimides to afford products **13a**–**d** in 45–60% yields with 2:1 to 3:1 dr (Scheme 9). The low diastereoselectivity is caused by the different R^2^/R^2’^ and R^3^/R^3’^groups which no longer have the same stereochemistry as that shown in Scheme 8.

### Double cycloadditions for bis[spirooxindole-pyrrolizidine] compounds

**Figure 3:**
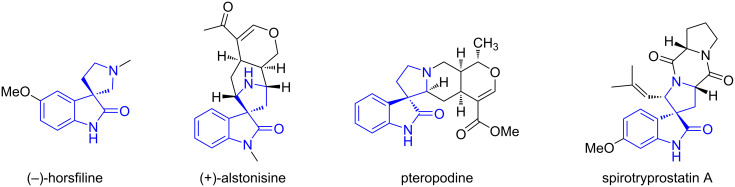
Biologically interesting spirooxindole-pyrrolizidines.

After completing the pseudo-five-component double cycloaddition reactions leading to polycyclic pyrrolizidines shown in Scheme 6 and Scheme 7, we then conducted similar reactions in order to synthesize spirooxindole-pyrrolidines. This unique ring skeleton exists in some natural products and biologically active compounds such as (−)-horsfiline, (+)-alstonisine, pteropodine and spirotryprostatin A (Figure 3) [70].

**Scheme 10:**
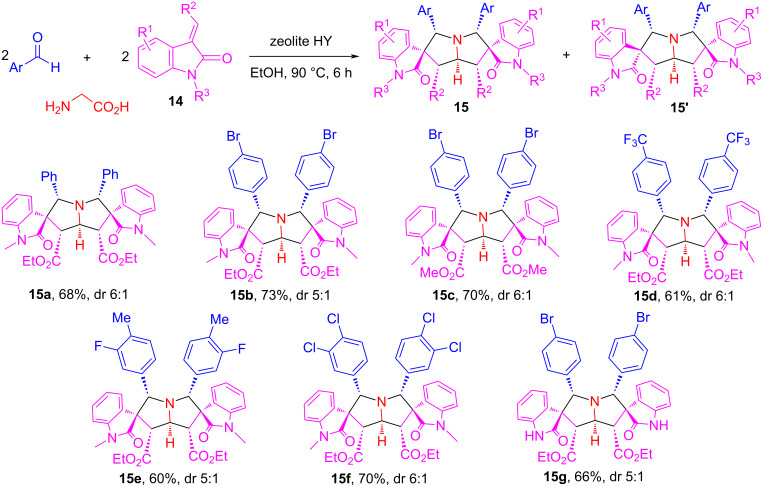
Double cycloadditions for the synthesis of bis[spirooxindole-pyrrolizidine]s.

**Scheme 11:**
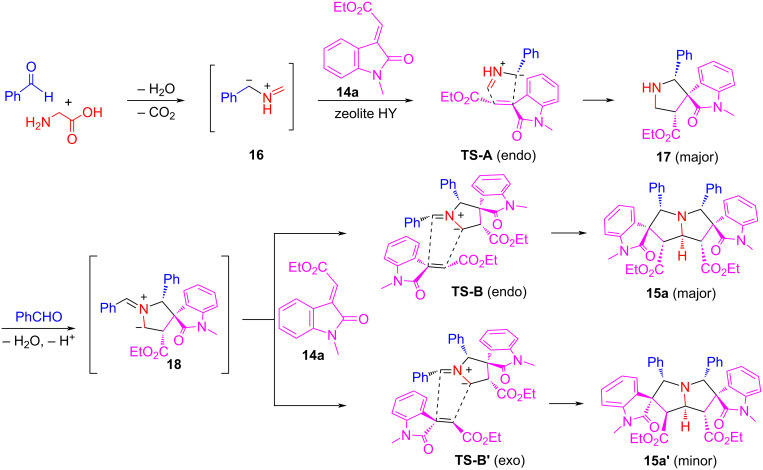
Mechanism for the diastereoselective synthesis of bis[spirooxindole-pyrrolizidine]s.

We expected that using olefinic oxindoles **14** as alkenes for the [3 + 2] cycloaddition could afford spirooxindole-pyrrolizidines. The method development revealed that recyclable zeolite HY acid is a good catalyst for the cycloaddition [70]. Thus, the zeolite HY-catalyzed reaction of glycine with two equiv each of arylaldehydes and olefinic oxindoles **14** in EtOH at 90 °C for 6 h gave bis[spirooxindole-pyrrolizidine] compounds **15a**–**g** in 60–73% yields with up to 6:1 dr (Scheme 10). It is worth noting that this pseudo-five-component reaction gives butterfly-shaped molecules which have a plane of symmetry. The stereochemistry of the products was confirmed by X-ray crystal structure and NMR analysis. The reaction mechanism shown in Scheme 11 suggests that a semi-stabilized AMY **16** generated from the reaction of glycine and arylaldehydes undergoes a [3 + 2] cycloaddition with **14a** via the favorable *endo*-transition state **A** to give spirooxindole-pyrrolizidine **17** which spontaneously reacts with another equiv of arylaldehyde to form ylide **18** in the presence of zeolite HY. The second [3 + 2] cycloaddition of **18** with **14a** affords product **15a** as a major product through an *endo-*cycloaddition and **15a’** as a minor diastereomeric product through an *exo*-cycloaddition.

### One-pot synthesis of triazolobenzodiazepines

**Scheme 12:**
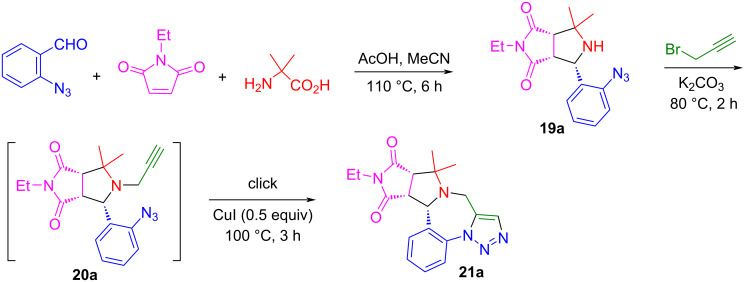
Stepwise synthesis of triazolobenzodiazepine **21a**.

Other than the multicomponent double cycloaddition reactions shown in the last section, we also utilized the first cycloaddition products for post-condensation reactions to generate new heterocyclic scaffolds. α-Substituted amino acids, such as 2-aminoisobutyric acid, could be used to block the second cycloaddition. Shown in Scheme 12 is a method development for the stepwise synthesis of triazolobenzodiazepines. The reaction of 2-azidobenzaldehyde, 2-aminoisobutyric acid and *N*-ethylmaleimide in MeCN under the catalysis of AcOH at 110 °C for 6 h afforded the monocycloaddition product **19a** in 93% LC yield [71]. The isolated compound **19a** was used for an *N*-propargylation to produce compound **20a** in 94% LC yield. The following Cu-catalyzed click reaction afforded triazolobenzodiazepine **21a** in 88% LC yield (Scheme 12).

**Scheme 13:**
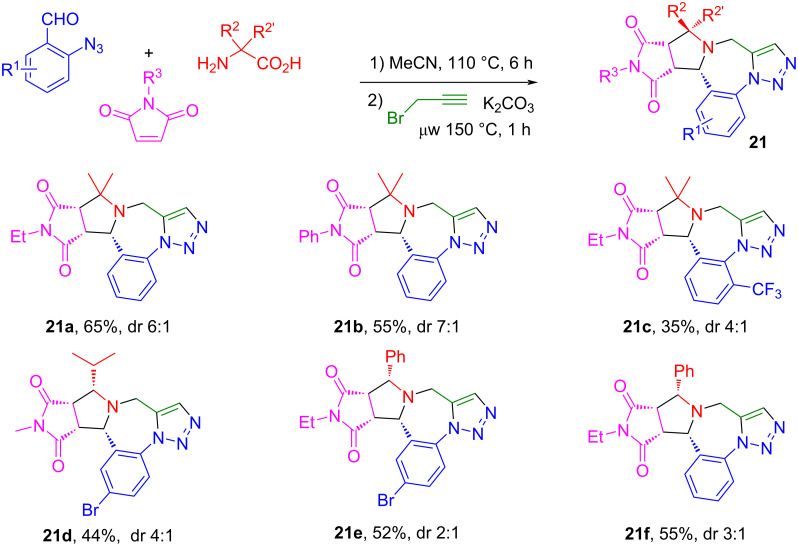
One-pot synthesis of triazolobenzodiazepines.

**Figure 4:**
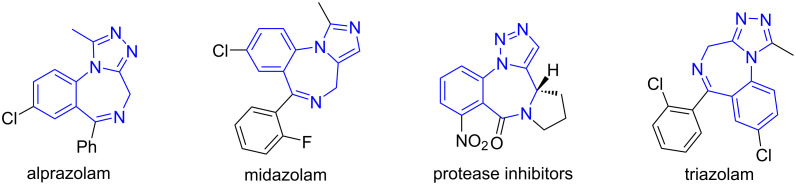
Bioactive triazolobenzodiazepine derivatives.

Our next goal was to convert the stepwise reaction process into a one-pot synthesis. After optimizing the reaction conditions, a one-pot two-step reaction was developed by the reaction of 2-azidobenzaldehydes, 2-substituted amino acids and maleimides with AcOH as a catalyst in MeCN at 110 °C for 6 h to afford the monocycloaddition compounds. Without isolation, the reaction mixtures were then used for the *N*-propargylation in the presence of K_2_CO_3_ under microwave heating at 110 °C for 1 h to give triazolobenzodiazepines **21a**–**f** in 35–65% yields with 2:1 to 7:1 dr (Scheme 13). Other than 2-aminoisobutyric acid, phenylglycine and valine with Ph or iPr groups could also be used for the synthesis of the monocycloaddition products for the post-condensation reactions. It is worth noting that in the one-pot synthesis involving an intramolecular click reaction, no Cu catalyst was used. A similar reaction sequence using stabilized AMYs was also reported from our lab [72]. The triazolobenzodiazepines obtained through this highly efficient one-pot synthesis have structure similarity with some drug molecules shown in Figure 4 [71].

### One-pot synthesis of pyrroloquinazolines and pyrrolobenzodiazepines

**Scheme 14:**
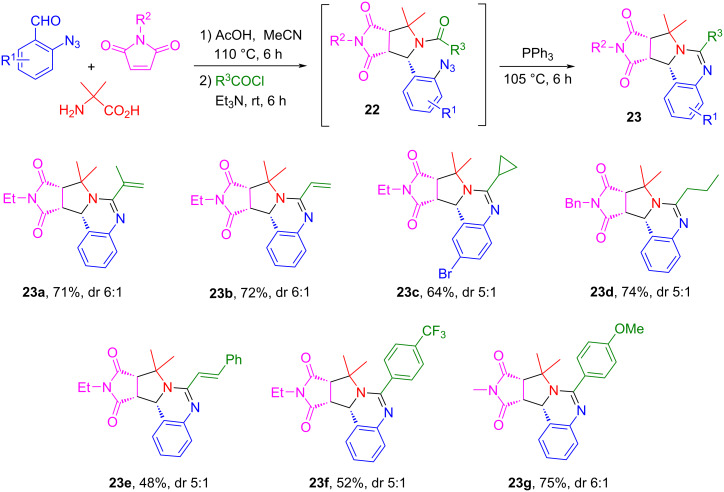
One-pot synthesis of tetrahydropyrroloquinazolines.

**Scheme 15:**
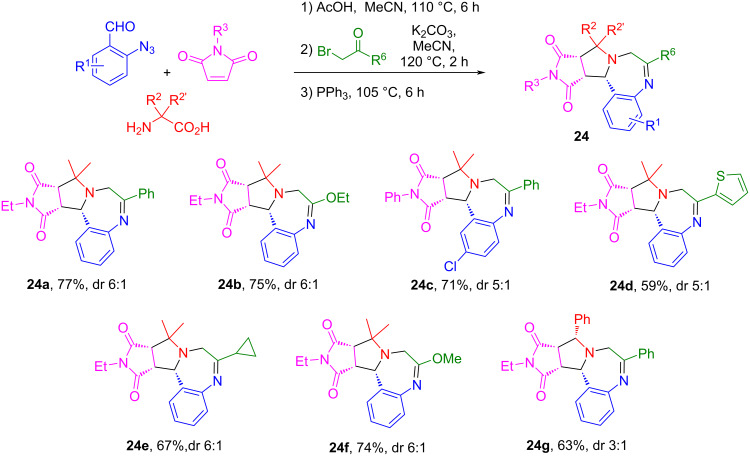
One-pot synthesis of tetrahydropyrrolobenzodiazepines.

**Figure 5:**
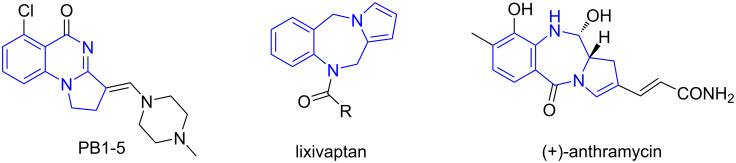
Bioactive pyrroloquinazolines and pyrrolobenzodiazepines.

We developed a 2-azidobenzladehyde-based reaction sequence including a one-pot [3 + 2] cycloaddition, *N*-acylation and Staudinger/aza-Wittig reactions for the construction of pyrroloquinazolines and pyrrolobenzodiazepines [73]. The AcOH-catalyzed reaction of 2-azidobenzaldehydes, α-substituted amino acids and maleimides in MeCN at 110 °C for 6 h afforded the corresponding monocycloaddition compounds followed by acylation to yield intermediates **22**. The subsequent sequential Staudinger/aza-Wittig reaction of intermediates **22** gave products **23a–g** in 48–75% yields with 5:1 to 6:1 dr (Scheme 14). This one-pot reaction could also be applied for the synthesis of pyrrolobenzodiazepines when using 2-bromoketones instead of the acid chlorides affording products **24a–g** in 59–77% yields with 3:1 to 6:1 dr (Scheme 15). The pyrroloquinazolines and pyrrolobenzodiazepines made by this route have structure similarity with bioactive compounds and natural products such as PB1-5 [74], lixivaptan, and (+)-anthramycin (Figure 5) [73].

### Stepwise synthesis of pyrrolo[2,1-*a*]isoquinolines

**Scheme 16:**
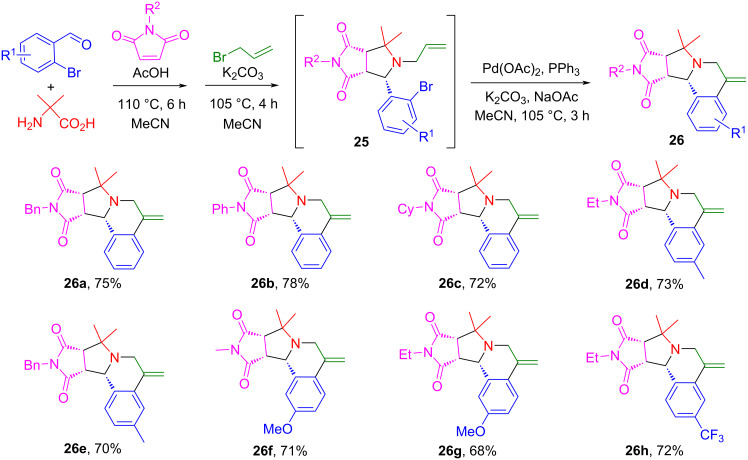
Stepwise synthesis of pyrrolo[2,1-*a*]isoquinolines.

**Figure 6:**
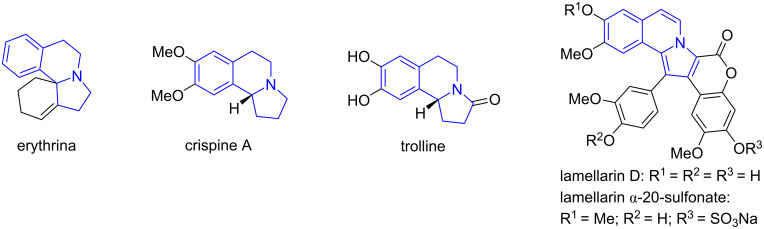
Bioactive pyrrolo[2,1-*a*]isoquinolines and hexahydropyrrolo[2,1-*a*]isoquinolines.

A stepwise synthesis involving [3 + 2] cycloaddition, *N*-allylation and Heck reactions has been developed for the synthesis of pyrrolo[2,1-*a*]isoquinolines. The reaction of 2-bromobenzaldehydes, 2-aminoisobutyric acid, and maleimides in MeCN under the catalysis of AcOH at 110 °C for 6 h afforded the cycloaddition products **26**. The purified intermediates were used for the one-pot *N*-allylation with allyl bromide to afford intermediate **25** followed by a Pd-catalyzed Heck reaction to give products **26** in 65–78% yields (Scheme 16) [75]. The pyrrolo[2,1-*a*]isoquinoline core installed by this route can be found in some natural products and synthetic compounds with antitumor, antibacterial, antiviral, antioxidizing, and other biological activities (Figure 6) [75].

### One-pot double annulations for the synthesis of tetrahydropyrrolothiazoles

**Figure 7:**
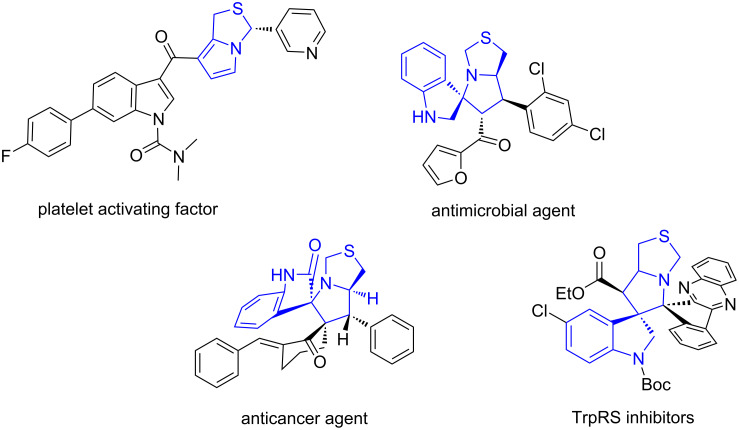
Bioactive tetrahydropyrrolothiazoles.

**Scheme 17:**
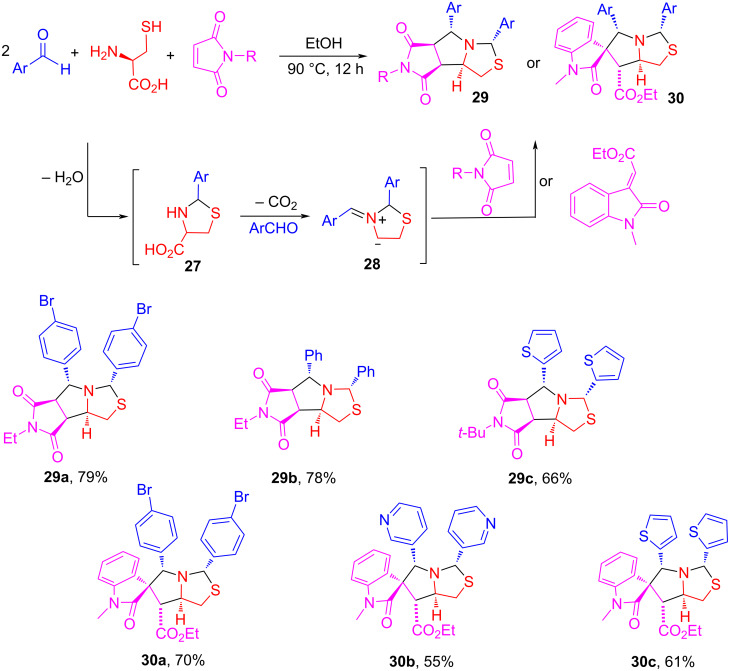
Pseudo-four-component reaction for the synthesis of tetrahydropyrrolothiazoles **29** and **30** (>4:1 dr).

**Scheme 18:**
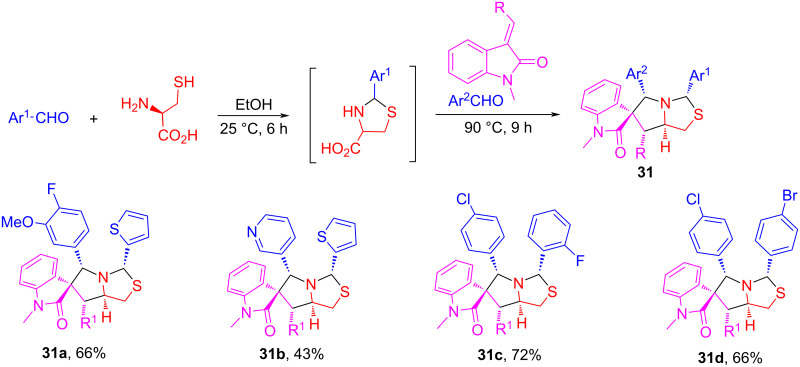
One-pot two-step synthesis of spirooxindole-pyrrolothiazoles **31** (>4:1 dr).

The unique tetrahydropyrrolothiazole and spiro[indole-tetrahydropyrrolothiazole] scaffolds are found in bioactive compounds such as those shown in Figure 7 [76–77]. Using cysteine as a key reactant, we developed a pseudo-four-component reaction for the synthesis of tetrahydropyrrolothiazole derivatives. The reaction of cysteine with two equiv of arylaldehydes and one equiv of maleimides in EtOH at 90 °C for 12 h afforded tetrahydropyrrolothiazoles **29** in 66–79% yields with up to 7:1 dr (Scheme 17) [76]. Using olefinic oxindoles to replace maleimides, the reactions gave spiro[indoline-tetrahydropyrrolothiazole] products **30** in 55–70% with greater than 4:1 dr [76]. The reaction mechanism suggests that the reaction of cysteine with arylaldehydes gives *N*,*S*-acetals **27** which convert to AMYs **28** after decarboxlyation. Cycloaddition of **28** with maleimides or olefinic oxindoles gives products **29** and **30**, respectively. The reactions could be carried out as a two-step synthesis using two different arylaldehydes to give products **31** in 43–72% yields with greater than 4:1 dr (Scheme 18). A similar reaction sequence based on a [3 + 2] cycloaddition of stabilized AMYs has been reported by our lab [78].

## Conclusion

The amino acid-based decarboxylative [3 + 2] cycloaddition reactions developed from our lab are summarized in this paper. The semi-stabilized *N*–H-type azomethine ylides derived from amino acids could be used for multicomponent, one-pot, and multistep reactions in the synthesis of heterocyclic compounds. The methods have advantages of using readily available starting materials, performing streamlined reactions, producing diverse product structures, and having high pot, atom, and step economy (PASE) [79–81] for the diversity-oriented synthesis (DOS) [82–88]. The work presented in this paper may also be helpful to understand the reaction mechanism and stereoselectivity of semi-stabilized *N*–H-type AMYs. We hope the new development for 1,3-dipolar cycloaddition chemistry can be used for the synthesis of bioactive heterocyclic compounds in medicinal and drug discovery programs.
